# A Doctor’s Diploma before a Jury

**Published:** 1883-10-20

**Authors:** 


### A DOCTOR'S DIPLOMA BEFORE A JURY.

The late Alexander H. Stephens used to tell, with great gusto, the following story, in which he and Robert Toombs figured :

A doctor named Royston had sued Peter Bennett for his bill, long overdue, for attending the wife of the latter. Alexander H. Stephens was on the Bennett side, and Robert Toombs, then in the United States Senate, was for the doctor. The doctor proved the number of his visits, their value according to local custom, and his own authority to do medical practice. Mr. Stephens told his client that the doctor had made out his case, and there was nothing wherewith to rebut or offset the claim, and the only thing left to do was to pay it. "No," said Peter, "I hired you to speak in my case, and now speak." Mr. Stephens told him there was nothing to say ; he had looked on to see that it was made out and it was. Peter was obdurate, and at last Mr. Stephens told Peter to make a speech himself, if he thought one could be made. "I will," said Peter, "if Bobby Toombs won't be too hard on me." Senator Toombs promised he would not, and Peter began.

'Gentlemen of the Jury, you and I is plain farmers, and if we don't stick together these ere lawyers and doctors will get the advantage of us. I ain't no lawyer or doctor, and I ain't no objections to them in their proper place, but they ain't farmers, gentlemen of the jury. Now this man, Royston, was no doctor, and I went for him to come and doctor my wife's sore leg, and he come and put some salve truck on to it, and some rags, but never done it a bit of good. Gentlemen of the jury, I don't believe he is a doctor, any way. There are doctors as is doctors, sure enough, but this man don't earn his money, and if you send for him, as Mrs. Sarah Atkinson did for a negro boy as was worth $1,000, he just kills him and wants you to pay it."

"I don't," thundered the doctor

"Did you cure him !" asked Peter, with the slow accents of a judge with a black cap on. The doctor was silent, and Peter proceeded :

"As I was saying, gentlemen of the jury, we farmers, when we sell our cotton, has got to give vally for the money we ask, and doctors ain't none too good to be put to the same rule. And I don't believe this ere Sam. Royston is a doctor no how."

"Look at my diploma, if you think I am no doctor."

"His diploma !" exclaimed the orator, with great contempt. "His diploma ! Gentlemen, that is a big word for printed sheepskin, and it don't make no doctor of the sheep as first wore it ; nor does it of the man as now carries it ; a good newspaper has more in it, and I pint out to ye that he ain't no doctor, at all."

The doctor was now in a fury, and screamed out :

"Ask my patients if I am not a doctor !"

"I asked my wife," retorted Peter. "She said she thought he was not."

"Ask my other patients," said the doctor.

This seemed to be the straw that broke the camel's back ; for Peter replied with look and tone of unutterable sadness : "That is a hard saying, gentlemen of the jury, and one that required me to die, or to have powers as I have hearn tell ceased to be exercised since the Apostles. Does he expect me to bring the angel Gabriel down to toot his horn before his time, and cry aloud,—'Awake, ye dead, and tell this court and jury your opinion of Sam Royston's practice?'

"Am I to go to the lonely churchyard and rap on the silent tomb and say to um as is at last at rest from physic and doctor's bills, 'Get up here, you, and state if you died a natural death, or was you hurried up some by doctors?' He says ask his patients, and gentlemen of the jury, *they are all dead!* Where is Mrs. Beasley's man, Sam? Go ask the worm in the graveyard, where he lies. Mr. Peak's woman, Sarah, was attended by him, and her funeral was appointed, and he, he, the doctor, had the corpse ready. Where is the likely Bill, as belonged to Mr. Mitchell? Now in glory, expressing his opinion of Royston's doctoring. Where is that baby gal of Harry Stephens? She is where doctors cease to trouble and infants are at rest. Gentlemen, he has eaten chicken enough at my house to pay for this salve. I found the rags, and I don't suppose he charges for making her worse, and even he don't pretend to charge for curing her, and I am humbly thankful he never give her nothing for her inwards, as he did his other patients, for something made them all die mighty sudden."

The applause was great. The doctor lost and Peter won.—*Presbyterian Observer.*

